# Changes in the Transcriptome Profiles of Human Amnion-Derived Mesenchymal Stromal/Stem Cells Induced by Three-Dimensional Culture: A Potential Priming Strategy to Improve Their Properties

**DOI:** 10.3390/ijms23020863

**Published:** 2022-01-13

**Authors:** Alessia Gallo, Nicola Cuscino, Flavia Contino, Matteo Bulati, Mariangela Pampalone, Giandomenico Amico, Giovanni Zito, Claudia Carcione, Claudio Centi, Alessandro Bertani, Pier Giulio Conaldi, Vitale Miceli

**Affiliations:** 1 Research Department, IRCCS ISMETT (Istituto Mediterraneo per i Trapianti e Terapie ad Alta Specializzazione), 90127 Palermo, Italy; agallo@ismett.edu (A.G.); ncuscino@ismett.edu (N.C.); fcontino@ismett.edu (F.C.); mbulati@ismett.edu (M.B.); gzito@ismett.edu (G.Z.); ccenti@ismett.edu (C.C.); pgconaldi@ismett.edu (P.G.C.); 2 Fondazione Ri.MED, 90127 Palermo, Italy; mpampalone@fondazionerimed.com (M.P.); gamico@fondazionerimed.com (G.A.); ccarcione@fondazionerimed.com (C.C.); 3 Department of Laboratory Medicine and Advanced Biotechnologies, IRCCS ISMETT (Istituto Mediterraneo per i Trapianti e Terapie ad Alta Specializzazione), 90127 Palermo, Italy; 4 Thoracic Surgery and Lung Transplantation Unit, IRCCS ISMETT (Istituto Mediterraneo per i Trapianti e Terapie ad Alta Specializzazione), 90127 Palermo, Italy; abertani@ismett.edu

**Keywords:** human amnion-derived mesenchymal stromal/stem cells, RNA sequencing, 3D priming, MSC spheroids, MSC therapeutic properties, regenerative medicine

## Abstract

Mesenchymal stromal/stem cells (MSCs) are believed to function in vivo as a homeostatic tool that shows therapeutic properties for tissue repair/regeneration. Conventionally, these cells are expanded in two-dimensional (2D) cultures, and, in that case, MSCs undergo genotypic/phenotypic changes resulting in a loss of their therapeutic capabilities. Moreover, several clinical trials using MSCs have shown controversial results with moderate/insufficient therapeutic responses. Different priming methods were tested to improve MSC effects, and three-dimensional (3D) culturing techniques were also examined. MSC spheroids display increased therapeutic properties, and, in this context, it is crucial to understand molecular changes underlying spheroid generation. To address these limitations, we performed RNA-seq on human amnion-derived MSCs (hAMSCs) cultured in both 2D and 3D conditions and examined the transcriptome changes associated with hAMSC spheroid formation. We found a large number of 3D culture-sensitive genes and identified selected genes related to 3D hAMSC therapeutic effects. In particular, we observed that these genes can regulate proliferation/differentiation, as well as immunomodulatory and angiogenic processes. We validated RNA-seq results by qRT-PCR and methylome analysis and investigation of secreted factors. Overall, our results showed that hAMSC spheroid culture represents a promising approach to cell-based therapy that could significantly impact hAMSC application in the field of regenerative medicine.

## 1. Introduction

In the last decade, many studies have highlighted the potential use of mesenchymal stromal/stem cells (MSCs) as a therapeutic tool to be applied in the field of regenerative medicine [1,2,3,4,5]. MSCs exhibit immunomodulatory, angiogenic, and regenerative capabilities, and these properties are mediated, at least in part, by paracrine mechanisms due to secretion of soluble factors [6,7,8,9,10,11,12,13,14]. Moreover, MSC abilities have raised them as a therapeutic tool in several clinical trials for the treatment of many disorders. However, the results obtained from these studies are controversial [15,16,17,18]. This phenomenon is probably related to the intrinsic properties of MSCs, which are derived from different sources [9,19]. Therefore, there is a need for improvement of MSC culture/production to enhance their therapeutic properties [3,20,21].

MSCs are found in several tissues, including bone marrow [22], adipose tissue [23], umbilical cord [24], and placenta [25], where these cells exhibit immunomodulatory [6,26,27,28], angiogenic [27,29,30], and antioxidative properties [31]. Regarding the source of MSCs, because of ethical issues and invasiveness for cell procurement, in recent years, increasing evidence supports the use of perinatal tissues, such as umbilical cord and placenta (e.g., amniotic membrane), as a useful source of MSCs [27,32,33]. Perinatal tissue shows several advantages, including the number of cells that can be easily obtained without any invasiveness.

It has been shown that MSC paracrine properties can be modulated by some preconditioning methods, including the growing of MSCs under three-dimensional (3D) culture conditions. Indeed, in response to 3D MSC priming/activation, the production of factors is switched toward a more anti-inflammatory and pro-trophic phenotype that results in an improvement in MSC therapeutic properties [21,34,35,36]. Our previous studies have shown that preconditioning of MSCs by 3D cultures enhances the secretion of functional factors. In particular, we revealed that conditioned medium (CM) derived from MSC spheroids was enriched with bioactive factors able to: (1) improve both wound healing and immunoregulatory capacity [27,37]; (2) enhance liver progenitor differentiation in an in vitro model of human liver organoids [8]; (3) attenuate the effects of cold ischemia–reperfusion injury in human alveolar epithelial cells [10]. Moreover, it has been demonstrated that when MSCs are grown as spheroids, they acquire stemness properties, increase cell survival, and improve their differentiation potential [34]. Therefore, spheroid formation can be considered as an optimization of MSC culture to enhance their therapeutic potential [20,21,38,39].

Recently, specific “omics” techniques were utilized to describe different biological processes. In particular, transcriptome analysis was used to understand MSC biological changes related to specific priming treatments (e.g., IL-17, IFN-γ, and hypoxia) [40,41] or to compare cells derived from different sources [42,43]. In recent years, total RNA sequencing (RNA-seq) using next-generation sequencing (NGS) platforms has greatly improved the analysis of whole transcriptomes, allowing for the individuation and quantification of the expression of a large number of genes. This technology can provide an unbiased analysis of the MSC activity with crucial insights into the affected cellular pathways. Thus, genome-wide molecular characterization of MSCs can allow the identification of their properties. This is very crucial to understand the appropriate culture methods to make MSCs suitable in the field of regenerative medicine.

In this work, we used RNA-seq analysis to provide a holistic view of transcriptome changes after 3D culture generation of human amnion-derived MSCs (hAMSCs). We observed extensive changes in gene expression profile (>9000 genes) following 3D cultures. Among 9000 deregulated genes, >4000 genes were upregulated, and Kyoto Encyclopedia of Genes and Genomes (KEGG) pathway analysis and Gene Ontology (GO) pathways analysis revealed that 30 upregulated genes were potentially implicated in crucial pathways involved in the regenerative processes. These findings highlight the importance of optimizing culture methods to maximize the therapeutic potential of MSCs. Understanding cellular responses following appropriate culture methods will help evaluate MSC application for specific biomedical applications.

## 2. Results

### 2.1. Isolation, Characterization, and Culture of hAMSCs

**Figure 1:**
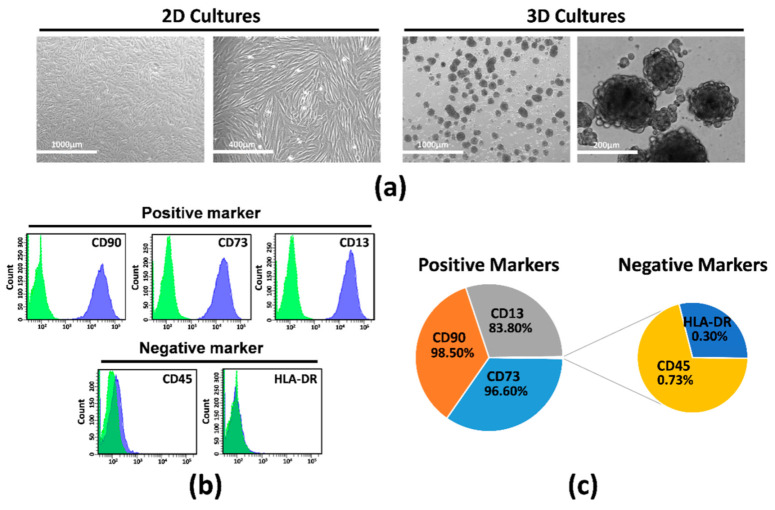
Human amnion mesenchymal stem cells (hAMSCs) grown as both monolayer and spheroids. (**a**) Representative DIC images of hAMSCs grown in monolayer (2D cultures) or as spheroids (3D cultures). (**b**) Representative images of flow cytometry analysis for quantification of both positive and negative surface markers in hAMSCs at Passage 0. Green represents isotype control, and blue represents stained cells. (**c**) Graphic depicts the percentage of each marker. DIC, differential interference contrast.

Primary cultures of MSCs were derived from the human amniotic membrane of the placenta, and adherent cells were expanded in vitro until Passage 2. Then, hAMSCs were grown in parallel in two-dimensional (2D) cultures (displaying morphologic and molecular characteristics that define MSCs) (Figure 1a–c) and in a suspended state (3D cultures), where cells spontaneously aggregated and formed compact multicellular spheroids (Figure 1a, right). Flow cytometry data showed positive expression of CD90 (98.50%), CD73 (96.60%), and CD13 (83.80%) and negative expression of hematopoietic lineage markers CD45 (0.73%) and HLA-DR (0.30%) (Figure 1b,c).

### 2.2. Gene Expression Profiles in 2D and 3D hAMSCs Revealed Enhanced Regenerative Properties of the hAMSC Spheroids

**Figure 2:**
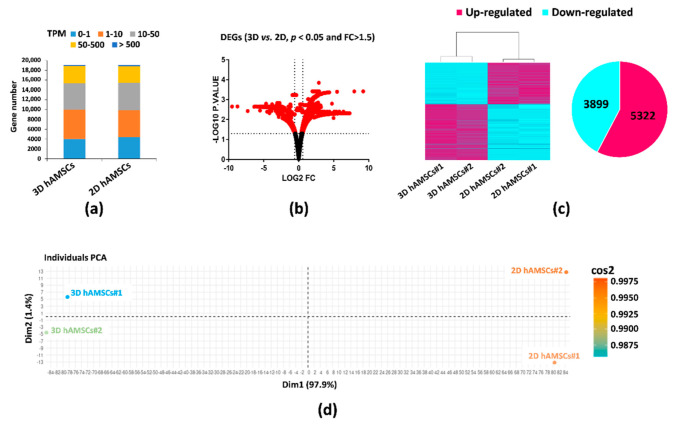
Gene expression profiles and differential expression in human amnion mesenchymal stem cells (hAMSCs) grown as both monolayer (2D) and spheroids (3D). (**a**) Gene expression distribution in both 2D and 3D hAMSCs. (**b**) Volcano plot analysis of differentially expressed genes (DEGs) in 3D vs. 2D hAMSCs (*p* < 0.05 and fold change >1.5). (**c**) Expression clusters (z-scores) of both up- and downregulated genes after volcano plot analysis in 2D and 3D hAMSCs. (**d**) Principal component analysis (PCA) of both 2D and 3D hAMSCs.

We used RNA-seq to examine gene expression changes in both 2D and 3D hAMSC cultures. We applied transcripts per kilobase million (TPM) to normalize and quantify each gene expression. About 19,000 genes were identified in both cultures (Figure 2a). In each sample, a relatively high expression (TPM ≥ 50) of 18.9% (2D hAMSCs) and 19.5% (3D hAMSCs) was detected (Figure 2a). Among these genes, the volcano plot (*p* < 0.05 and FC > 1.5) revealed 9221 significant differentially expressed genes (DEGs) (Figure 2b), where 3899 were downregulated, and 5322 were upregulated in 3D hAMSCs compared to 2D hAMSCs (Figure 2c). Interestingly, principal component analysis (PCA) and the heat map of DEGs clustered the samples into two distinct groups based on 2D or 3D cultures (Figure 2c,d).

**Figure 3:**
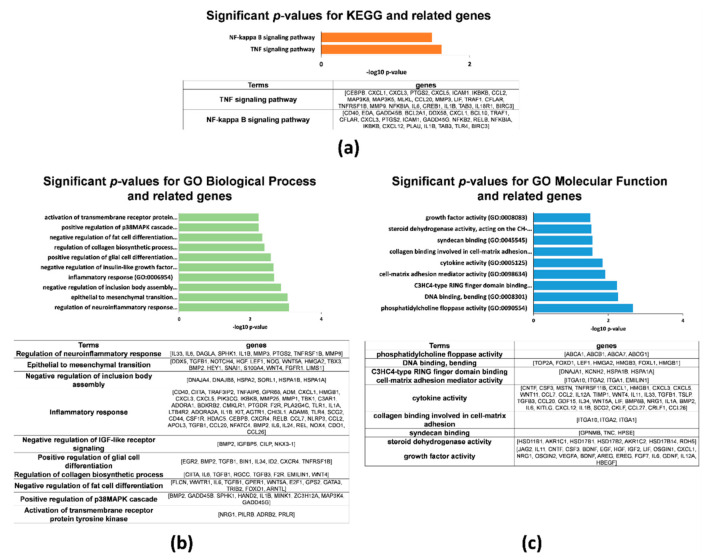
Functional enrichment analysis on KEGG pathways and Gene Ontology (GO) terms from the top 3000 upregulated genes in 3D vs. 2D hAMSCs. (**a**) Significant KEGG functional pathways (*p* < 0.05). (**b**) Significant GO terms of associated biological processes (*p* < 0.05). (**c**) Significant GO terms of associated molecular function (*p* < 0.05).

Both KEGG and GO enrichment analysis revealed that, among the top 3000 upregulated genes found in 3D hAMSCs, many DEGs were linked to immunomodulation, proliferation/differentiation, and angiogenesis. For example, KEGG analysis showed that the only two significant enriched pathways were the “TNF signaling pathway” and the “NF-kappa B signaling pathway” (Figure 3a). Many of the GO terms (ranked by *p*-value) associated with DEGs for 3D hAMSCs were also related to aspects of immune regulation, cellular growth, differentiation, and angiogenesis, including the terms “regulation of neuroinflammatory response”, “inflammatory response”, “positive regulation of glial cell differentiation”, “positive regulation of p38MAPK cascade”, “cytokine activity”, and “growth factor activity” (Figure 3b,c).

**Figure 4:**
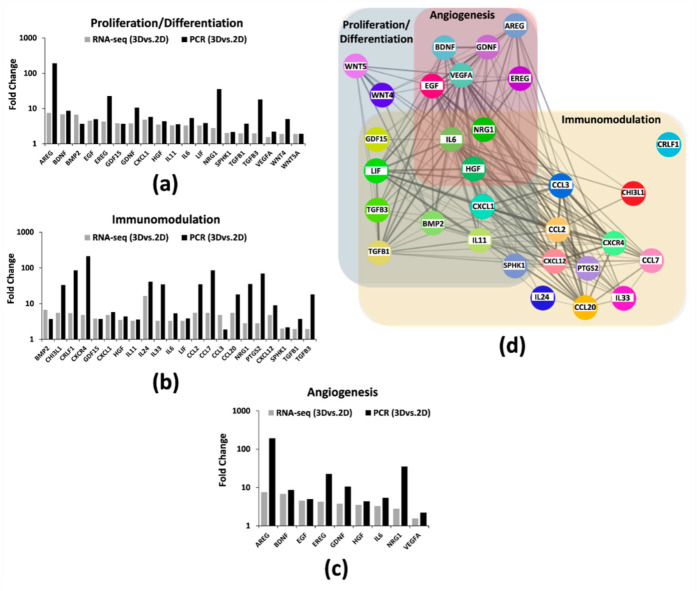
Transcriptomic analysis revealed 3D-induced hAMSC bioactivity. Upregulated genes involved in (**a**) proliferation/differentiation, (**b**) immunomodulatory, and (**c**) angiogenesis pathways were validated using qRT-PCR, indicating a similar trend. (**d**) Protein–protein interaction (PPI) network relied on experimentally derived functional and/or structural evidence, including the upregulated genes found.

After pathway enrichment analysis, we used qRT-PCR to validate RNA-seq results, detecting 30 randomly selected DEGs. In particular, as shown in Figure 4a–c, 30 genes we found upregulated by RNA-seq in 3D hAMSCs were significantly correlated with qRT-PCR results, indicating the reliability and accuracy of RNA-seq expression/analysis. The STRING database was used to construct a protein–protein interaction (PPI) map of the 30 aforementioned genes. After, using Cytoscape software, we identified a network with three protein clusters belonging to immune modulation, proliferation/differentiation, and angiogenesis pathways (Figure 4d).

### 2.3. Spheroid Formation of hAMSCs Induced Changes in Methylation Status and Increased the Production of Bioactive Factors

Using bisulfite sequencing analysis, we examined the methylation status of the 30 upregulated genes found in 3D hAMSCs by both RNA-seq and qRT-PCR (Table 1).

**Figure 5:**
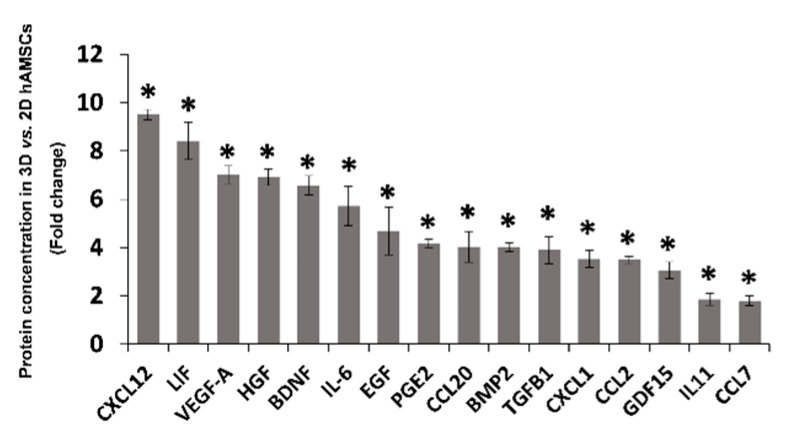
Protein secretion analysis of differentially methylated genes in 2D vs. 3D hAMSCs. * *p* < 0.05 vs. 2D hAMSCs.

We observed a clear decrease in the methylation levels (all sites) of sixteen genes in 3D hAMSCs compared to 2D hAMSCs (Table 1, bold). Thus, we analyzed the protein expression of the above-described cytokines/chemokines and growth factors in medium conditioned by both 2D and 3D cultures. We detected variable levels of those proteins. In particular, as shown in Figure 5, compared with hAMSCs 2D cultures, 3D spheroids showed significantly enhanced secretory activity for C-X-C motif chemokine ligand 12 (CXCL12), leukemia inhibitory factor (LIF), vascular endothelial growth factor A (VEGF-A), hepatocyte growth factor (HGF), brain-derived neurotrophic factor (BDNF), interleukin-6 (IL6), epidermal growth factor (EGF), prostaglandin E2 (PGE2), chemokine (C-C motif) ligand 20 (CCL20), bone morphogenetic protein 2 (BMP2), transforming growth factor beta 1 (TGFB1), C-X-C motif chemokine ligand 1 (CXCL1), C-C motif chemokine ligand 2 (CCL2), growth differentiation factor 15 (GDF15), interleukin-11 (IL11), and chemokine (C-C motif) ligand 7 (CCL7) (9.5-, 8.4-, 7-, 6.9-, 6.6-, 5.7-, 4.7-, 4.2-, 4-, 4-, 3.9-, 3.5-, 3.5-, 3-, 1.8-, and 1.8-fold, respectively).

## 3. Discussion

Many studies have widely demonstrated that MSCs represent one of the most promising cell products to treat numerous disorders in the field of regenerative medicine [44,45,46]. Indeed, the therapeutic action of MSCs is being currently investigated in several clinical trials (1356 studies registered at ClinicalTrials.gov) for the treatment of many disorders, including immune, kidney, cardiovascular, neurodegenerative, lung, liver, and orthopedics diseases. On the other hand, MSCs have been shown to have moderate or poor efficacy, and the results from different clinical trials are controversial [15,16,17]. Moreover, the variability and heterogeneity inter-donor or among tissue sources have been proved [47,48,49]. Overall, those challenges indicate an urgent need to optimize the therapeutic use of MSCs or to enhance MSC capabilities.

In the last decade, in order to promote MSC therapeutic use, different priming strategies have been used according to the injured tissue/organ to be targeted. For instance, the priming of MSCs with proinflammatory cytokines and 3D cultures has been mainly tested to modulate the inflammation and to stimulate angiogenesis in injured tissues [6,27,39,50]. Three-dimensional growth of MSCs represents an easy and more physiologic culture that usefully changes MSC phenotype. Many papers have shown the ability of 3D culture to potentiate the therapeutic properties of MSCs, making them more suitable as cell therapeutic products [3,21,34]. Santos et al. demonstrated that a 3D culture model of umbilical cord mesenchymal stromal cells can be used to prime the secretome of those cells for potential clinical applications [38]. Moreover, it has been shown that dynamic 3D techniques for in vitro MSC culture allows the formation of viable compact cellular spheroids with therapeutic properties [20]. Our works also revealed that CM derived from 3D MSCs contained an increased amount of immunosuppressive and growth factors compared to 2D cultures [27], and 3D MSC-CM is capable of attenuating ischemia–reperfusion injury in an in vitro model of the lung [10]. Recently, we have also shown that the paracrine component derived from MSC spheroids was capable of improving in vitro the differentiation of human liver progenitor cells [8]. In that case, our work may suggest that MSCs could also stimulate resident adult stem cells in injured tissue, improving tissue regeneration and function recovery. Therefore, 3D cultures of MSCs can be considered as an advance for the optimization of MSC culture to enhance their therapeutic potential.

To date, the molecular mechanisms contributing to the improvement of MSC spheroid therapeutic properties remain unclear. Several studies have been performed to find gene expression similarity/variability among MSCs derived from different sources [42,51,52,53]. Moreover, transcriptome analysis was used to study gene expression variations related to specific priming treatments, such as IL-17, IFN-γ, and hypoxia [40,41]. However, as far as we know, no study has already investigated, by the transcriptome approach, the molecular changes underlying the 3D growth of MSCs. Many scientific data focused on the expression differences within MSCs derived from distinct origins [54,55,56], but, in the field of MSC therapy, it has become very important also to investigate the differences among MSCs derived from the same tissue. In particular, functional differences could come from different methodological approaches, including chemical or physical treatment of MSCs during culture prior to their use.

**Figure 6:**
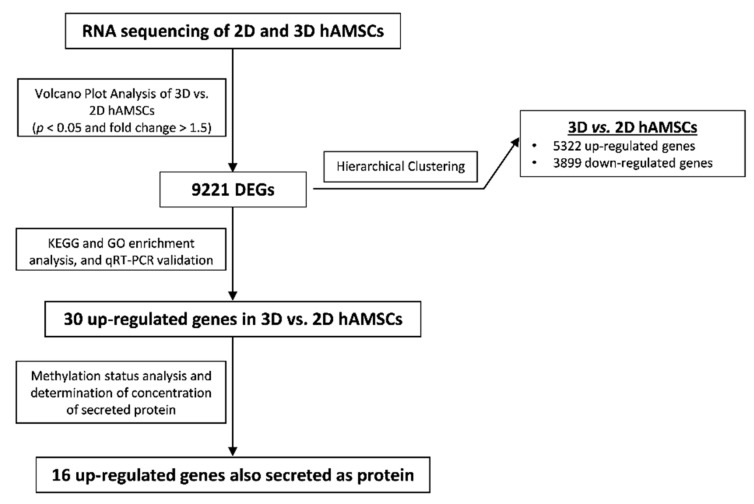
Gene screening process. Data analysis workflow in both 2D and 3D hAMSCs.

To address this, we performed RNA-seq to analyze both 2D and 3D cultures of MSCs derived from the amniotic membrane of the human placenta (hAMSCs) and investigate their molecular variations influencing their therapeutic properties. As shown in Figure 6 (data analysis workflow), to obtain more reliable data, we performed various approaches for the gene screening analysis.

In particular, through RNA-seq analysis, we first revealed that 9221 genes were deregulated, 3899 downregulated, and 5322 upregulated after 3D culture (Figure 2c). Then, we used both KEGG and GO enrichment analyses and identified that several DEGs were related to pathways implicated in regenerative medicine, such as immunomodulation, proliferation/differentiation, and angiogenesis (Figure 3). We used qRT-PCR analysis to validate RNA-seq results and identified 30 DEGs belonging to the above-mentioned pathways (Figure 4). Secondly, on those genes, we further analyzed their methylation status and found significant differences on only 16 genes (Table 1, bold). Finally, we analyzed the protein expression of these genes, and we found that CXCL12, LIF, VEGF-A, HGF, BDNF, IL6, EGF, PGE2, CCL20, BMP2, TGFB1, CXCL1, CCL2, GDF15, IL11, and CCL7 were more secreted by 3D hAMSCs compared to 2D hAMSCs.

Many researchers have previously demonstrated the pleiotropic role of the factors we found upregulated in hAMSC spheroids. It has been shown that BDNF, VEGF-A, IL6, and HGF stimulate angiogenesis both in vitro and in vivo [30,57,58,59,60], and EGF, VEGF-A, and HGF were shown to be significantly higher in MSC-treated ischemic tissue, mediating neovascularization effects [61]. The factors described above have also been shown to have immunomodulatory capabilities. Indeed, although we do not yet know the detailed mechanisms by which those factors suppress/modulate immune responses, it has been revealed that MSCs are able to affect immune responses secreting soluble factors, such as TGFβ1, HGF, PGE2, IL6, LIF, and GDF15 [6,27,62,63]. Moreover, it was demonstrated that CCL2, CXCL12, and BMP2 produced by MSCs were able to affect M1 polarization macrophages in favor of the M2 phenotype [64,65], and IL11 was able to induce Th2 polarization of human CD4^+^ T cells [66]. Interestingly, the above-described factors have also been implicated in tissue regeneration processes. In particular, it was shown that MSC-derived CM was enriched in factors, including IL6, HGF, and VEGF-A, and this secretome was able to induce liver regeneration [5,67]. Gothelf and collaborators demonstrated that the CM derived from neurotrophic factor-secreting MSCs was enriched with BDNF, VEGF-A, and HGF and has been used effectively to have protective effects in several animal models of neurodegenerative diseases [68]. EGF stimulates the growth of numerous epidermal and epithelial tissues [69], and activation of TGF-β is also involved in the recruitment of stem/progenitor cells in tissue regeneration/remodeling processes [70]. Furthermore, it has been proven that LIF, which plays a crucial role in blastocyst implantation, is able to regulate some regenerative processes after injury in several tissues [71]. Oka et al., in an in vivo model, showed that both GDF15 and IL6 cooperate to induce survival of transplanted brown adipose tissues [72]. Finally, high levels of IL6, CCL2, and CXCL1 in the wound microenvironment were associated with tissue repair [73].

To date, by omics techniques, very few works have analyzed the molecular mechanisms underlying MSC priming activation. The characterization of MSC therapeutic properties is problematic due to the variability in the production of different bioactive molecules according to interdonor variation and depending on their origin [49,74]. Moreover, perinatal MSCs, including amniotic-derived MSCs, possess a higher therapeutic potential [49]. Therefore, there is the need to find an easily accessible source of MSCs without invasiveness and to optimize detailed protocols to improve the standardization of MSCs production with effective therapeutic properties.

In our work, we used multiple methodological approaches (RNA-seq, qRT-PCR, methylome analysis, and evaluation of protein secretion) to analyze the gene/protein variations among hAMSCs grown as spheroids, which were associated with potential therapeutic properties. We observed that 3D culture conditions generated a large amount of DEGs belonging to the crucial pathways involved in the regenerative processes, such as immunomodulation, proliferation/differentiation, and angiogenesis. Moreover, we revealed that both genetic and epigenetic variations potentially contribute to functional activation of 3D hAMSCs. MSCs derived from the human amniotic membrane of the placenta are a new advantageous source of MSCs, and 3D culture priming of those cells, differently to other priming systems using exogenous factors (IL-17, IFN-γ, IL1, TNFα) could represent a useful and natural method to enhance MSC therapeutic properties in a low manipulation setting. This is very crucial in order to implement MSC cellular therapies to be applied in the field of regenerative medicine.

## 4. Materials and Methods

### 4.1. Isolation, Culture, and Phenotypic Characterization of Human Amnion-Derived Mesenchymal Stromal/Stem Cells

MSCs were isolated from the amnion of the human term placenta of healthy donors. Written informed consent and the procedure were approved by ISMETT’s Institutional Research Review Board. Informed consent was obtained from each donor. After separation between amnion and chorion, amniotic membrane was cut into small pieces and decontaminated in three different solutions containing: (1) 2.5% Esojod (Esoform, Rovigo, Italy); (2) 500 U/mL penicillin, 500 mg/mL streptomycin, 12.5 mg/mL amphotericin B, and 1.87 mg/mL cefamezin (Pfizer, Milan, Italy); (3) 100 U/mL penicillin and 100 mg/mL streptomycin. Amniotic fragments were digested for 9 min at 37 °C in HBSS (Lonza, Basel, Switzerland) containing 2.5 U/mL dispase (Corning, New York, NY, USA) and then maintained for 5 min in RPMI 1640 (Thermo Fisher Scientific, Waltham, MA, USA). Afterward, the amniotic fragments were further digested with 0.94 mg/mL collagenase A (Roche, Mannheim, Germany) and 20 mg/mL DNase (Roche, Mannheim, Germany) for 2.5 h at 37 °C. The cell suspension obtained was filtered with both 100 μm and 70 μm cell strainers (BD Falcon, San Jose, CA, USA), pelleted, and resuspended in RPMI for cell counting. Harvested cells were cultured in polystyrene culture dishes (Corning, New York, NY, USA) at 37 °C, 5% CO_2_, in Chang Medium (Irvine Scientific, Santa Ana, CA, USA). To obtain hAMSCs at different passages, the cells were plated at a density of 1 × 10^4^/cm^2^, and after reaching confluence, adherent cells were trypsinized and then subcultured until Passages 3–5. HAMSCs were phenotypically characterized by cytofluorimetric analysis for positive markers (CD90, CD73, and CD13) and negative markers (CD45 and HLA-DR) (BD Biosciences, San Jose, CA, USA). Analysis was performed using the FACSCanto II flow cytometer (Becton Dickinson, Franklin Lakes, NJ, USA) and FACSDiva 8.0.1 (Becton Dickinson, Franklin Lakes, NJ, USA) software.

### 4.2. Mesenchymal Stromal/Stem Cell Spheroid Cultures

HAMSCs at the second passage were cultured as spheroids in a 6-well ultralow attachment plate (Corning, New York, NY, USA), which facilitates spheroid formations and their maintenance. HAMSC spheroids were maintained in DMEM serum-free medium at 5% CO_2_ and 37 °C.

### 4.3. Conditioned Media Preparation

To collect CM from 2D culture, after the cells reached 90–95% confluence, the medium was replaced with serum-free DMEM medium, and the cells were grown for 2 days for CM collection. For 3D cultures, first, we observed the initial spheroid formation for 1 day, after the medium was changed, conditioned for 2 days, and finally collected. The supernatant from each culture was centrifuged and frozen at −80 °C until use.

### 4.4. Gene Expression Profiling

HAMSC total RNA was extracted with the RNeasy Mini Kit and treated with DNase according to the manufacturer’s instructions (QIAGEN, Hilden, Germany). Then, it was converted to complementary DNA using the high-capacity cDNA kit (Thermo Fisher Scientific, Waltham, MA, USA). Gene expression was analyzed with the QuantStudio™ 7 Pro Real-Time PCR System (Thermo Fisher Scientific, Waltham, MA, USA) using 18S ribosomal RNA (Hs03928985_g1) as housekeeping. We used TaqMan assays for *AREG* (Hs00950669_m1), *BDNF* (Hs02718934_s1), *BMP2* (Hs00154192_m1), *CCL2* (Hs00234140_m1), *CCL20* (Hs00355476_m1), *CCL3* (Hs00234142_m1), *CCL7* (Hs00171147_m1), *CHI3L1* (Hs01072228_m1), *CRLF1* (Hs00191064_m1), *CXCL1* (Hs00236937_m1), *CXCL12* (Hs03676656_mH), *CXCR4* (Hs00607978_s1), *EGF* (Hs01099990_m1), *EREG* (Hs00914313_m1), *GDF15* (Hs00171132_m1), *GDNF* (Hs01931883_s1), *HGF* (Hs00300159_m1), *IL11* (Hs01055414_m1), *IL24* (Hs01114274_m1), *IL33* (Hs04931857_m1), *IL6* (Hs00174131_m1), *LIF* (Hs01055668_m1), *NRG1* (Hs01101538_m1), *PTGS2* (Hs00153133_m1), *SPHK1* (Hs00184211_m1), *TGFB1* (Hs00998133_m1), *TGFB3* (Hs01086000_m1), *VEGF-A* (Hs00900055_m1), *WNT4* (Hs01573505_m1), *WNT5A* (Hs00998537_m1). Reactions were run in duplicate, and calculation of the relative levels of expression was performed according to the comparative Ct-method.

### 4.5. Protein Expression Analysis

The concentration of cytokine and growth factors (CXCL12, LIF, VEGF-A, HGF, BDNF, IL6, EGF, CCL20, BMP2, TGFB1, CXCL1, CCL2, GDF15, and CCL7) in each CM (hAMSCs grown in both 2D and 3D cultures) were determined using magnetic bead technology from Luminex™ with the ProcartaPlex Multiplex protein assays (Affymetrix, Santa Clara, CA, USA) according to the manufacturer’s instructions. The levels of IL11 and PGE-2 were determined using the Human IL-11 Quantikine ELISA Kit and PGE2 Parameter Assay Kit (R&D Systems, Minneapolis, MN, USA), respectively. The concentration of each factor was calculated from standard curves.

### 4.6. RNA-Seq, Library Construction, Sequencing, and Analysis

Total RNA from 2D and 3D hAMSCs was isolated with RNeasy Micro Kit according to the manufacturer’s instructions (QIAGEN, Hilden, Germany). Concentration and quality of RNA were determined using the Qubit 2.0 Fluorometer (Life Technologies, Carlsbad, CA, USA) and 4200 TapeStation System (Agilent Technologies, Santa Clara, CA, USA). Libraries were generated from 1 µg of RNA derived from 2D and 3D hAMSC cultures. Poly-A-enriched strand-specific libraries were generated with the TruSeq mRNA V2 sample preparation kit with Ribo-Zero Gold (Illumina, San Diego, CA, USA). The quality and yield of the prepared libraries were assessed using Qubit 2.0 Fluorometer (Life Technologies, Carlsbad, CA, USA) and 4200 TapeStation System (Agilent Technologies, Santa Clara, CA, USA). Sequencing was performed on a NextSeq™ 550 (Illumina, San Diego, CA, USA) with 2 × 76 cycles, following the manufacturer’s instructions. Quality control checks of the sequencing raw data were conducted with FastQC (v0.11.9, Babraham Institute). Both low-quality read removal and adapter-trimming were performed with trimmomatic (v0.32) [75]. The remaining reads were mapped to human reference genome hg19 with STAR (v2.7.0) [76], and transcript abundances were measured with RSEM (v1.3.3) [77]. We normalized the gene expression level by transcripts per kilobase million (TPM). To identify differentially expressed genes (DEGs), we set the fold change ≥1.5 or ≤0.6666. DEGs were hierarchically clustered using an average linkage algorithm and a “Euclidean distance” for the distance measure using R functions (v4.1.2). The same software was used to perform principal component analysis (PCA).

### 4.7. Bisulfite Genomic Sequencing Analysis of DNA Methylation

We used bisulfite genomic sequencing as a method of DNA methylation analysis. Briefly, gDNA from 2D and 3D hAMSCs was isolated with the AllPrep DNA/RNA Micro Kit, according to the manufacturer’s instructions (QIAGEN, Hilden, Germany). Concentration and quality of gDNA were determined using the Qubit 2.0 Fluorometer (Life Technologies, Carlsbad, CA, USA) and 4200 TapeStation System (Agilent Technologies, Santa Clara, CA, USA). The SureSelect Target Enrichment System (Agilent Technologies, Santa Clara, CA, USA) was used to identify epigenetic changes in 2D and 3D hAMSC cultures. All samples were sequenced using the same workflow. Briefly, 1 μg of gDNA was sheared using the Covaris sonicator (Covaris, Woburn, MA, USA) to yield 170–230 bp DNA fragments. The DNA fragments were end-repaired, 3′-adenylated, and further ligated with methylated primers. Following hybridization to biotinylated, plus-strand DNA-complementary RNA library “baits” and streptavidin bead enrichment, captured DNA was bisulfite-converted using the EZ DNA Methylation Gold Kit (Zymo Research, Irvine, CA, USA). Subsequently, DNA samples were PCR-amplified using barcoded indexed primers to allow for multiplexing. Quality and yield of the DNA sample libraries were assessed using Qubit 2.0 Fluorometer (Life Technologies, Carlsbad, CA, USA) and 4200 TapeStation System (Agilent Technologies, Santa Clara, CA, USA). The pooled libraries were sequenced with the NextSeq™ 550 (Illumina, San Diego, CA, USA).

### 4.8. Whole-Genome Bisulfite Sequencing Data Mapping and Quality Analysis

On raw data from both bisulfite sequencing converted and non-bisulfite sequencing converted, we performed quality analysis with FastQC (v0.11.9, Babraham Institute, Babraham, UK), and we trimmed with Trim Galore (v0.6.5, Babraham Institute). After, mapping was carried out with Bismark (v0.22.3, Babraham Institute) to hg19 for the human genome. We used SeqMonk software (v1.48.0, Babraham Institute) to quantify the percentage of CpG methylation values.

### 4.9. Pathway Enrichment Analysis

To find both KEGG and GO terms enriched in the defined gene sets, we used the Enrichr web tool [78]. For figures, we only reported the significant top 10 ranked terms. After gene validation, for map visualization, pathway enrichment analysis results were analyzed by STRING web tool [79] and then interpreted in Cytoscape 3.9.0 [80].

### 4.10. Statistics

All values were expressed as mean ± SD. Statistical analysis was performed using GraphPad Prism 6.0 (GraphPad Software, San Diego, CA, USA). The Student’s t-test was used to compare data. Differences were considered statistically significant at *p* < 0.05.

## 5. Conclusions

In conclusion, our work investigated a transcriptomic portrait of hAMSCs in which a significant change in transcriptomic profile was observed in response to 3D culture. The profiling of genes that we found provided new insights related to 3D molecular changes of a new source of MSCs derived from the human amniotic membrane of the placenta. Conventional 2D culture has been seen as a limitation in the wider use of MSC-based therapies, and the development of 3D culture has become a current technological challenge. Indeed, MSCs cultured in monolayer possess limited reproducibility/scalability. Moving MSC culture from a 2D to a 3D suspension culture system allows culturing these cells in a more physiological microenvironment that can potentiate their therapeutic properties. Modulation of the transcriptome/secretome of MSCs is a crucial step toward achievement of the full therapeutic potential of MSCs. Our data revealed that hAMSCs grown in 3D culture represent a promising prime method to improve hAMSC therapeutic properties and highlight the importance of increasing our understanding of MSC biology under different culture/priming methods to optimize their potential therapeutic use.

## Figures and Tables

**Table 1:** Methylation levels (all sites) of upregulated genes in 2D and 3D hAMSCs.

Gene	Chromosome	Start	End	Description	2D hAMSCs (%)	3D hAMSCs (%)
*AREG*	4	75480629	75490486	Amphiregulin	74.70	73.60
*BDNF*	**11**	**27676440**	**27743605**	**Brain-derived neurotrophic factor**	**14.08**	**10.24**
*BMP2*	**20**	**6748311**	**6760910**	**Bone morphogenetic protein 2**	**7.32**	**4.45**
*CCL2*	**17**	**32582313**	**32584222**	**Chemokine (C-C motif) ligand 2**	**24.41**	**12.93**
*CCL20*	**2**	**228678558**	**228682272**	**Chemokine (C-C motif) ligand 20**	**84.90**	**77.06**
*CCL3*	17	34415602	34417515	Chemokine (C-C motif) ligand 3	42.86	56.67
*CCL7*	**17**	**32597240**	**32599261**	**Chemokine (C-C motif) ligand 7**	**45.19**	**39.34**
*CHI3L1*	1	203148059	203155877	Chitinase 3-like 1	68.58	76.02
*CRLF1*	19	18704037	18717660	Cytokine receptor-like factor 1	17.79	17.66
*CXCL1*	**4**	**74735110**	**74736959**	**Chemokine (C-X-C motif) ligand 1**	**18.06**	**11.30**
*CXCL12*	**10**	**44793038**	**44881941**	**Chemokine (C-X-C motif) ligand 12**	**30.98**	**26.83**
*CXCR4*	2	136871919	136875735	Chemokine (C-X-C motif) receptor 4	7.47	6.95
*EGF*	**4**	**110834040**	**110933422**	**Epidermal growth factor**	**60.13**	**56.20**
*EREG*	4	75230860	75254468	Epiregulin	26.62	26.19
*GDF15*	**19**	**18496968**	**18499986**	**Growth differentiation factor 15**	**20.99**	**16.99**
*GDNF*	5	37812779	37839788	Glial cell-derived neurotrophic factor	16.70	16.05
*HGF*	**7**	**81328322**	**81399754**	**Hepatocyte growth factor**	**50.33**	**41.92**
*IL11*	**19**	**55875757**	**55881814**	**Interleukin 11**	**26.80**	**21.64**
*IL24*	1	207070788	207077484	Interleukin 24	71.90	70.12
*IL33*	9	6215805	6257983	Interleukin 33	60.55	68.69
*IL6*	**7**	**22765503**	**22771621**	**Interleukin 6**	**19.70**	**12.45**
*LIF*	**22**	**30636436**	**30642840**	**Leukemia inhibitory factor**	**48.59**	**41.73**
*NRG1*	8	31496902	32622548	Neuregulin 1	37.85	36.44
*PTGS2*	**1**	**186640923**	**186649559**	**Prostaglandin-endoperoxide synthase 2**	**32.49**	**27.00**
*SPHK1*	17	74372742	74383941	Sphingosine kinase 1	14.60	13.79
*TGFB1*	**19**	**41836813**	**41859831**	**Transforming growth factor, beta 1**	**20.05**	**16.55**
*TGFB3*	14	76424442	76449334	Transforming growth factor, beta 3	20.21	19.32
*VEGF-A*	**6**	**43737921**	**43754224**	**Vascular endothelial growth factor A**	**25.56**	**21.88**
*WNT4*	1	22446461	22470462	Wingless-type MMTV integration site family, member 4	43.70	48.77
*WNT5A*	3	55499743	55523973	Wingless-type MMTV integration site family, member 5A	28.81	31.67

Bold indicates a significant decrease in the methylation levels in 3D compared to 2D hAMSCs.

## Data Availability

The datasets used and analyzed are available from the corresponding author on reasonable request.

## Abbreviations

2D	Two-dimensional
3D	Three-dimensional
CM	Conditioned medium
DEGs	Differentially expressed genes
GO	Gene Ontology
hAMSCs	Amnion-derived mesenchymal stromal/stem cells
KEGG	Kyoto Encyclopedia of Genes and Genomes
MSCs	Mesenchymal stromal/stem cells
NGS	Next-generation sequencing
PCA	Principal component analysis
PPI	Protein–protein interactions
RNA-seq	RNA sequencing
TPM	Transcripts per kilobase million
